# Collateral ligament strain is linearly related to coronal lower limb alignment: A biomechanical study

**DOI:** 10.1002/ksa.12340

**Published:** 2024-06-27

**Authors:** Christian Peez, Luise Maria Hägerich, Felix Ruhl, Matthias Klimek, Thorben Briese, Johannes Glasbrenner, Adrian Deichsel, Michael J. Raschke, Christoph Kittl, Elmar Herbst

**Affiliations:** ^1^ Department of Trauma, Hand and Reconstructive Surgery University Hospital Münster Münster Germany

**Keywords:** collateral ligament strain, coronal lower limb alignment, individualized collateral ligament reconstruction, lateral collateral ligament, superficial medial collateral ligament

## Abstract

**Purpose:**

The purpose of this study was to analyse the influence of coronal lower limb alignment on collateral ligament strain.

**Methods:**

Twelve fresh‐frozen human cadaveric knees were used. Long‐leg standing radiographs were obtained to assess lower limb alignment. Specimens were axially loaded in a custom‐made kinematics rig with 200 and 400 N, and dynamic varus/valgus angulation was simulated in 0°, 30°, and 60° of knee flexion. The changes in varus/valgus angulation and strain within different fibre regions of the collateral ligaments were captured using a three‐dimensional optical measuring system to examine the axis‐dependent strain behaviour of the superficial medial collateral ligament (sMCL) and lateral collateral ligament (LCL) at intervals of 2°.

**Results:**

The LCL and sMCL were exposed to the highest strain values at full extension (*p* < 0.001). Regardless of flexion angle and extent of axial loading, the ligament strain showed a strong and linear association with varus (all Pearson's *r* ≥ 0.98; *p* < 0.001) and valgus angulation (all Pearson's *r* ≥ −0.97; *p* < 0.01). At full extension and 400 N of axial loading, the anterior and posterior LCL fibres exceeded 4% ligament strain at 3.9° and 4.0° of varus, while the sMCL showed corresponding strain values of more than 4% at a valgus angle of 6.8°, 5.4° and 4.9° for its anterior, middle and posterior fibres, respectively.

**Conclusion:**

The strain within the native LCL and sMCL was linearly related to coronal lower limb alignment. Strain levels associated with potential ultrastructural damages to the ligaments of more than 4% were observed at 4° of varus and about 5° of valgus malalignment, respectively. When reconstructing the collateral ligaments, an additional realigning osteotomy should be considered in cases of chronic instability with a coronal malalignment exceeding 4°–5° to protect the graft and potentially reduce failures.

**Level of Evidence:**

There is no level of evidence as this study was an experimental laboratory study.

## Graphical abstract

Native collateral ligament strain was linearly related to coronal lower limb alignment so that strain levels associated with potential ultrastructural damages to the ligaments of more than 4% were observed at 4° of varus and about 5° of valgus malalignment, respectively. In cases of chronic instability with coronal malalignment exceeding 4°–5°, realigning osteotomy should be considered in addition to collateral ligament reconstruction.

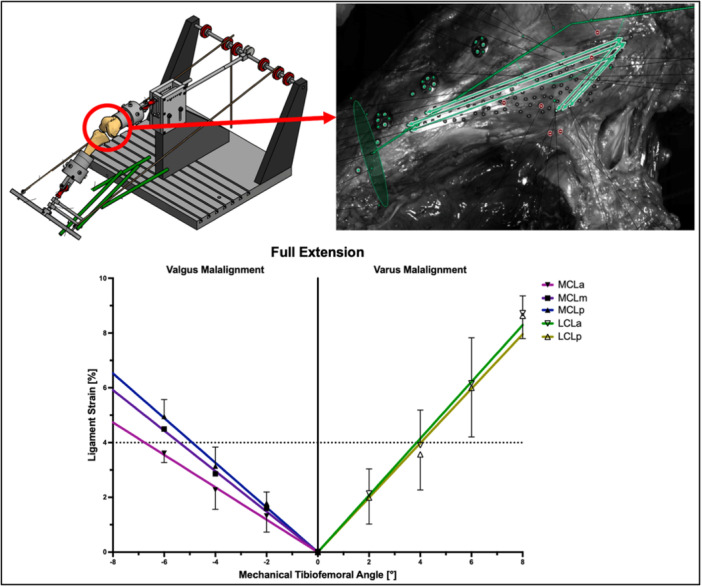

**Abbreviations**
LCL: lateral collateral ligament
MFA: mechanical femoral axis
MTA: mechanical tibial axis
mTFA: mechanical tibiofemoral angle
PFL: popliteofibular ligament
PLT: popliteus tendon
POL: posterior oblique ligament
sMCL: superficial medial collateral ligament

## INTRODUCTION

Despite advances in the surgical treatment of knee collateral ligament injuries, recurrent instability after lateral and medial collateral ligament reconstruction remains a major concern, with graft failure rates ranging from 10% to 37% [30, 31, 32]. While the influence of surgical technique [3, 9, 10, 16], graft isometry [4, 18, 22, 49] and overlooked collateral ligament injuries with subsequent rotatory knee instability [13, 16, 48] have been extensively investigated, bony deformities have been widely neglected as a potential cause of treatment failures [7, 14, 46]. In this context, however, it has been assumed that coronal lower limb malalignment may cause failure of lateral and medial collateral ligament reconstructions in about one‐third of the cases [5, 32, 46].

As the knee bears about three times the body weight during normal gait [43], the grafts of the reconstructed medial and lateral collateral ligaments (LCLs) are subjected to high forces, especially in case of varus or valgus malalignment [1, 36, 43]. Over time, these repetitive excess forces across the malaligned knees may stretch out the grafts, resulting in secondary insufficiency or failure [33, 38]. As recent biomechanical studies have shown a relieving effect of realigning osteotomies on the knee ligaments [17, 23, 28], soft tissue balancing becomes more aware as a key factor in knee reconstructive surgeries to reduce failures [7, 14, 20]. However, to date, there are no threshold values if and when a realigning osteotomy is indicated as an adjunct to a medial or LCL reconstruction to protect the graft and potentially improve patient‐reported outcomes.

Therefore, the aim of this study was to analyse the influence of coronal lower limb alignment on collateral ligament strain of the native knee to define thresholds for combined realignment and reconstructive procedures. It was hypothesized that (1) the strain within the native ligaments is linearly associated with lower limb alignment and (2) a lower limb malalignment of more than 5° is associated with strain values above 4%.

## MATERIALS AND METHODS

Six pairs (*n* = 12) of fresh‐frozen human cadaveric knees age (79.7 ± 5.7 years, three females and three males) without history of previous bony or ligamentous injuries of the lower limbs were obtained from the local anatomical institute (Institute of Anatomy and Molecular Neurobiology, University of Muenster, Muenster, Germany). The specimens were dissected and biomechanically tested under the necessary permission of the Institutional Ethics Committee of the University of Muenster (File number 2020‐181‐f‐S).

### Lower limb alignment analysis

**Figure 1:**
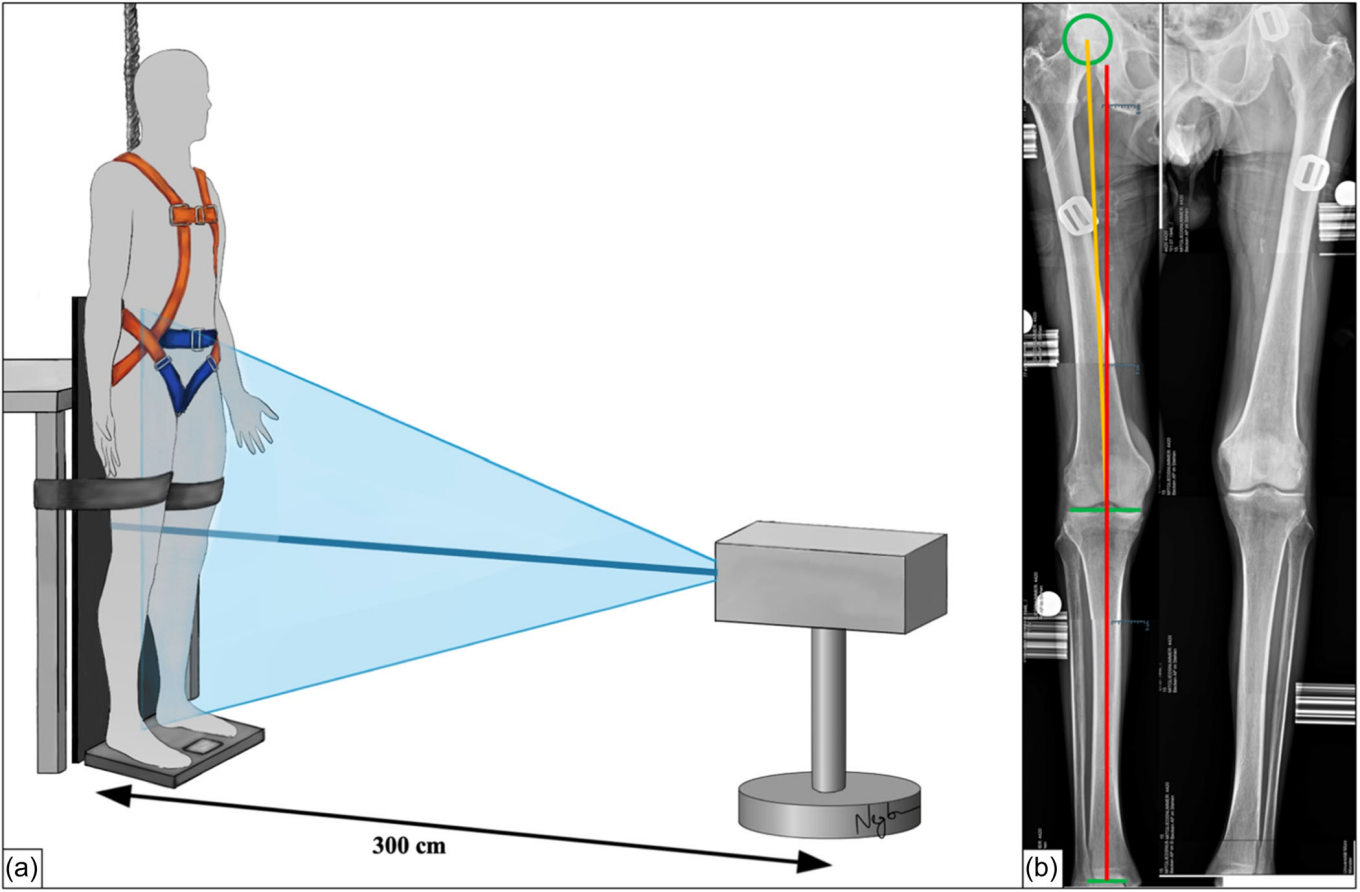
Coronal limb alignment analysis. (a) Schematic illustration of the long‐leg standing radiographs as previously described [12]. The full‐body donors were secured with a harness in an upright bipedal standing position and placed on a scale to ensure full weight bearing. The pelvis and mid‐thighs were strapped to the X‐ray rig to prevent uncontrolled bending of the knee joints during stance. (b) Example of an anteroposterior whole‐leg standing radiograph showing a constitutional valgus as indicated by a negative mechanical tibiofemoral angle of −2°. Mechanical tibial axis = red line; mechanical femoral axis = orange line.

Prior to biomechanical testing, full‐length anterior–posterior standing radiographs of the lower limbs were obtained. Once the specimens were thawed at room temperature for 72 h, the human bodies were secured in an upright position using a harness to achieve a bipedal stance and placed on a scale to ensure full weight bearing. To prevent uncontrolled bending of the knee joints during stance, the pelvis and the mid‐thigh were strapped to a custom‐made X‐ray rig. The beam was set 300 cm from the specimen at the level of the knee joint to reduce beam distortion and ensure an equidistant trajectory from both the femur and tibia. For calibration, a reference circle with a diameter of 25 mm was suspended at the level of the knee joint (Figure 1a). Whole‐leg standing radiographs were considered adequate if the patellae were centred on the femoral condyles [35].

Coronal lower limb alignment was analysed as described by Paley et al. [35]. For this, the anatomic landmarks were defined as follows: (1) The mechanical tibial axis (MTA) was drawn as a line from the midpoint of the tibial plateau to the midpoint of the proximal talus. (2) The mechanical femoral axis (MFA) was defined as a line from the centre of the femoral head to the centre of the distal femoral joint line, which represented the tangent to the most distal part of the femoral condyles. Coronal alignment was then quantified as the mechanical tibiofemoral angle (mTFA) between the MTA and MFA according to Strecker [45], while a negative mTFA refers to valgus alignment and a positive mTFA implies varus alignment. All measurements were performed by a senior orthopaedic consultant (E. H.) (Figure 1b).

### Specimen preparation

Upon completion of lower limb alignment analysis, the knee joints were harvested by cutting the femur and tibia at a distance of 250 mm proximal and distal to the joint line and stored at −20°C until biomechanical testing. After thawing at room temperature for 24 h, the fibula was secured to the tibia in its anatomical position by a 3.5‐mm tricortical position screw. Then, an intramedullary stainless steel rod with a diameter of 12.0 mm was cemented into the femoral and tibial shaft using polyurethane bone cement (RenCast®; Gößl & Pfaff, Karlskron, Germany), which was locked for rotational stability with two 3.5‐mm bicortical screws. After removal of the skin and subcutaneous tissue, the medial and the lateral sides were anatomically dissected to identify the collateral ligaments. For the medial side, layer I was dissected to expose the femoral and tibial attachments of the superficial medial collateral ligament (sMCL) and posterior oblique ligament (POL) [37, 39]. At the lateral side, the iliotibial band was longitudinally divided at its posterior border to expose the posterolateral corner consisting of the LCL, popliteus tendon and popliteofibular ligament [8, 29].

**Figure 2:**
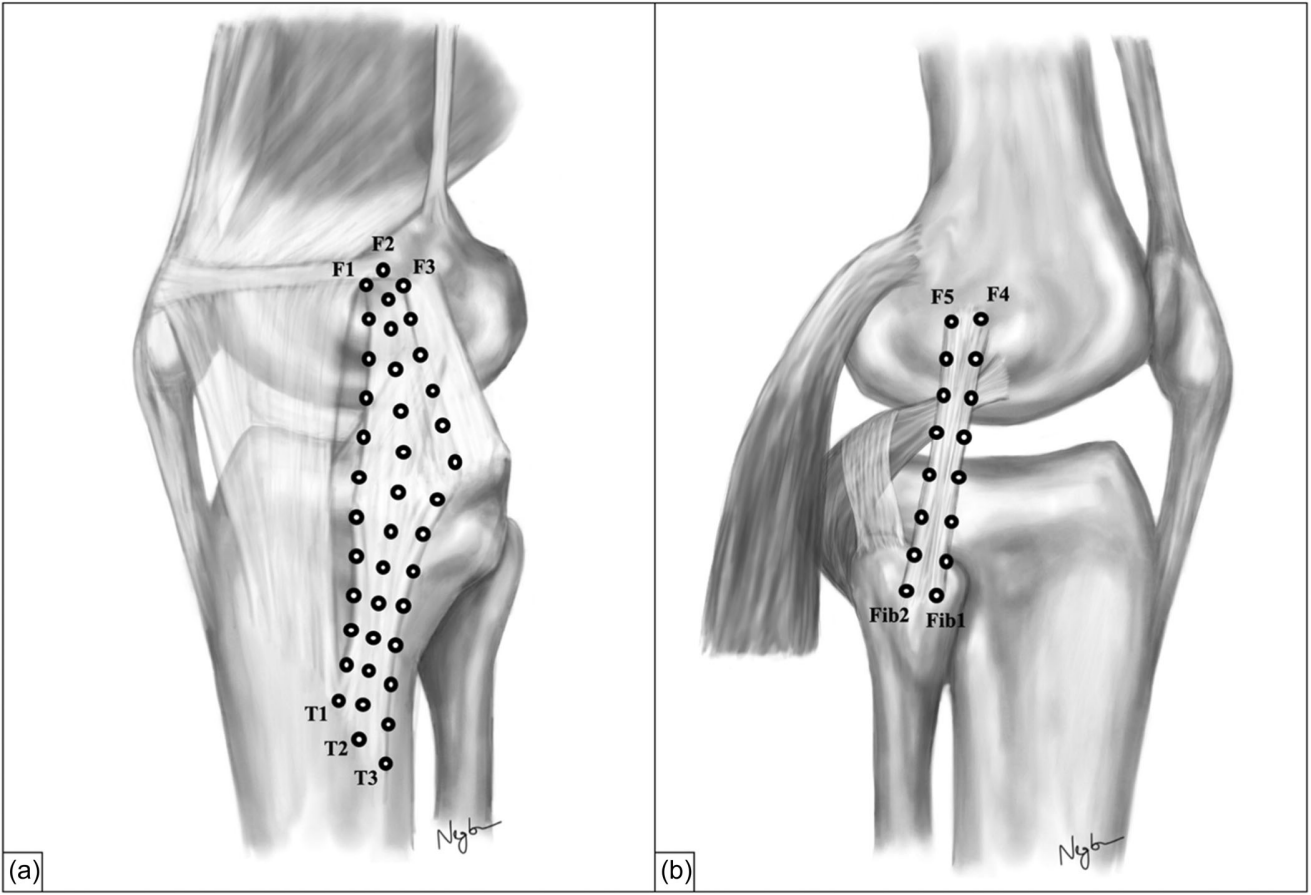
Illustration of the marked collateral ligaments. Each marker combination represents a distinct anatomical part of the superficial medial collateral ligament and lateral collateral ligament, respectively. (a) Anterior fibres of the sMCL (T1 – F1), middle fibres of the sMCL (T2 – F2), posterior fibres of the sMCL (T3 – F3). (b) Anterior fibres of the LCL (Fib1 – F4), posterior fibres of the LCL (Fib2 – F5).

After dissection, reflective optical markers with a diameter of 2.7 mm were evenly placed along the tibial and femoral shaft as well as on the sMCL and LCL for motion tracking. Care was taken to mark the most distal and proximal parts of the collateral ligaments to account for the entire length of the ligaments. At the medial side, the anterior, middle and posterior fibre regions of the sMCL were marked according to a standardized protocol [22] (Figure 2a). At the lateral side, only two fibre regions of the LCL could be labelled due to its width of 4–5 mm [24]. Accordingly, the anterior and posterior fibres of the LCL were marked with optical markers [8, 29] (Figure 2b).

**Figure 3:**
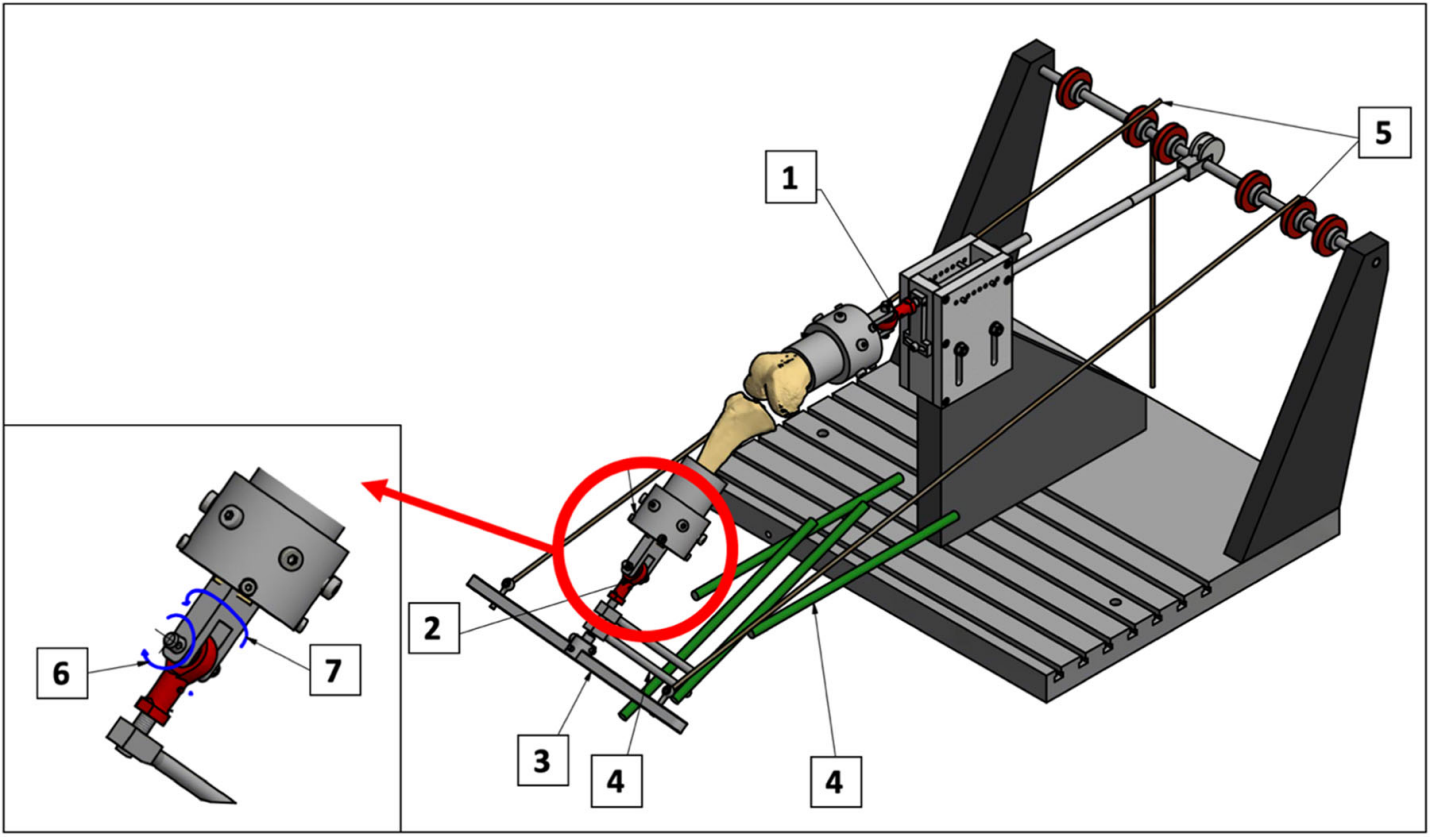
Schematic illustration of the custom‐made kinematics rig. The femur (1) and tibia (2) were connected via rotating/tilting joints to the open chain extension rig. The specimens were axially loaded with 200 and 400 N using a tibial‐based loading bar (3) and hanging weights via a pulley system (5). Guide rods (4) enabled flexion of the knee. The rotation joints (6) allowed an unrestricted varus/valgus angulation of the specimen, while the tilting joints (7) were restricted to ±10° of tibiofemoral rotation. Dynamic varus/valgus stress was simulated at 0°, 30° and 60° knee flexion until reaching the elastic limits of the collateral ligaments.

After preparation, each specimen was mounted in a custom‐made kinematics rig. The femoral intramedullary rod was secured via two connection rods to prevent rotation so that the posterior femoral condyle axis was aligned parallel to the rig. Tibially, the intramedullary rod was connected to a loading bar, which allowed axial loading of the knee joint using hanging weights via a pulley system. Both the femur and tibia were connected to a rotation/tilting joint to individually correct the previously determined constitutional varus/valgus deformity to a straight mechanical axis (Figure 3). To confirm the neutral alignment after mounting the knees to the kinematics rig, short anteroposterior radiographs were taken of each specimen at full extension and under 400 N of axial loading. The mismatch between short anteroposterior and full‐length standing radiographs was taken into account by correlating the anatomical TFA of the short radiographs with the anatomical TFA of the full‐length radiographs so that the corresponding mTFA could be determined to confirm a straight mechanical leg axis as indicated by an mTFA of 0° ± 1°.

### Biomechanical testing

The specimens were axially loaded in the kinematics rig with 200 and 400 N using hanging weights via a pulley system. In one knee of each specimen, the sMCL was tested, followed by the LCL. The other knee was tested in a reverse order. Depending on the order of testing, five cycles of varus/valgus stress were applied manually until reaching the elastic limits of the collateral ligaments to simulate a dynamic varus/valgus under axial loads of 200 and 400 N. The elastic limits of either the medial and lateral ligamentous structures were felt as a firm endpoint. Testing was performed for each specimen at 0°, 30° and 60° knee flexion.

### Data acquisition and analysis

Changes in the length of the medial and lateral ligamentous structures as well as the varus/valgus angulation were captured using an optical three‐dimensional measuring system (Aramis SRX, GOM GmbH), operating at a maximum acceptance error of ±0.004 mm [41]. For each testing condition, the relative movements between the optical markers were evaluated at the initial stage with a straight mechanical leg axis corresponding to an mTFA of 0° and at intervals of 2° of dynamic varus/valgus to calculate the length change of the different fibre regions of the sMCL and LCL. Calibration of the optical measuring system was performed at full extension of the knees with neutral rotation and before correcting lower limb alignment to rule out any contractures and laxities of the collateral ligament in subjects with constitutional varus/valgus. The absolute length of each tested tibiofemoral attachment combination was used to calculate the strain within the sMCL and LCL [(length change/absolute length at 0° mTFA and full extension) × 100%].

### Statistical analysis

Statistical analysis was performed using Prism (version 10, GraphPad Software). Data are presented as means ± standard deviation (SD). Normality of data distribution was tested and proved using the Shapiro–Wilk test. To compare the strain across varus/valgus angles and loading conditions with the data at 0° of coronal alignment, a two‐way repeated‐measures analysis of variance with a post hoc Bonferroni test was performed. Pearson's correlation and linear regression were obtained to calculate the relationship between the mTFA and the strain within the sMCL and LCL. Correlation coefficients between 0.40 and 0.69 were considered as ‘moderate’, between 0.70 and 0.89 as ‘strong’ and between 0.90 and 1.00 as ‘very strong’ [40]. Since strain levels of more than 4% are associated with microdamages to ligaments and tendons and therefore considered clinically relevant [42]; a critical threshold analysis was performed to determine the fibre region‐specific varus/valgus cut‐off value across the loading conditions and flexion angles at which 4% ligament strain was observed. Level of significance was set at 0.05.

An a priori power analysis was performed using G*Power‐2 software (University of Düsseldorf) [11]. Based on means and SDs from previous studies investigating the length change pattern of the sMCL [22, 49], it was assumed that a sample size of 8 would allow the detection of 1% changes in ligament strain (effect size 0.9) with 95% power at the significance level of *p* < 0.05.

## RESULTS

The mTFA ranged from −1.9° (valgus) to +7.9° (varus) (mean: 2.0 ± 3.3°).

### Effect of varus malalignment

**Figure 4:**
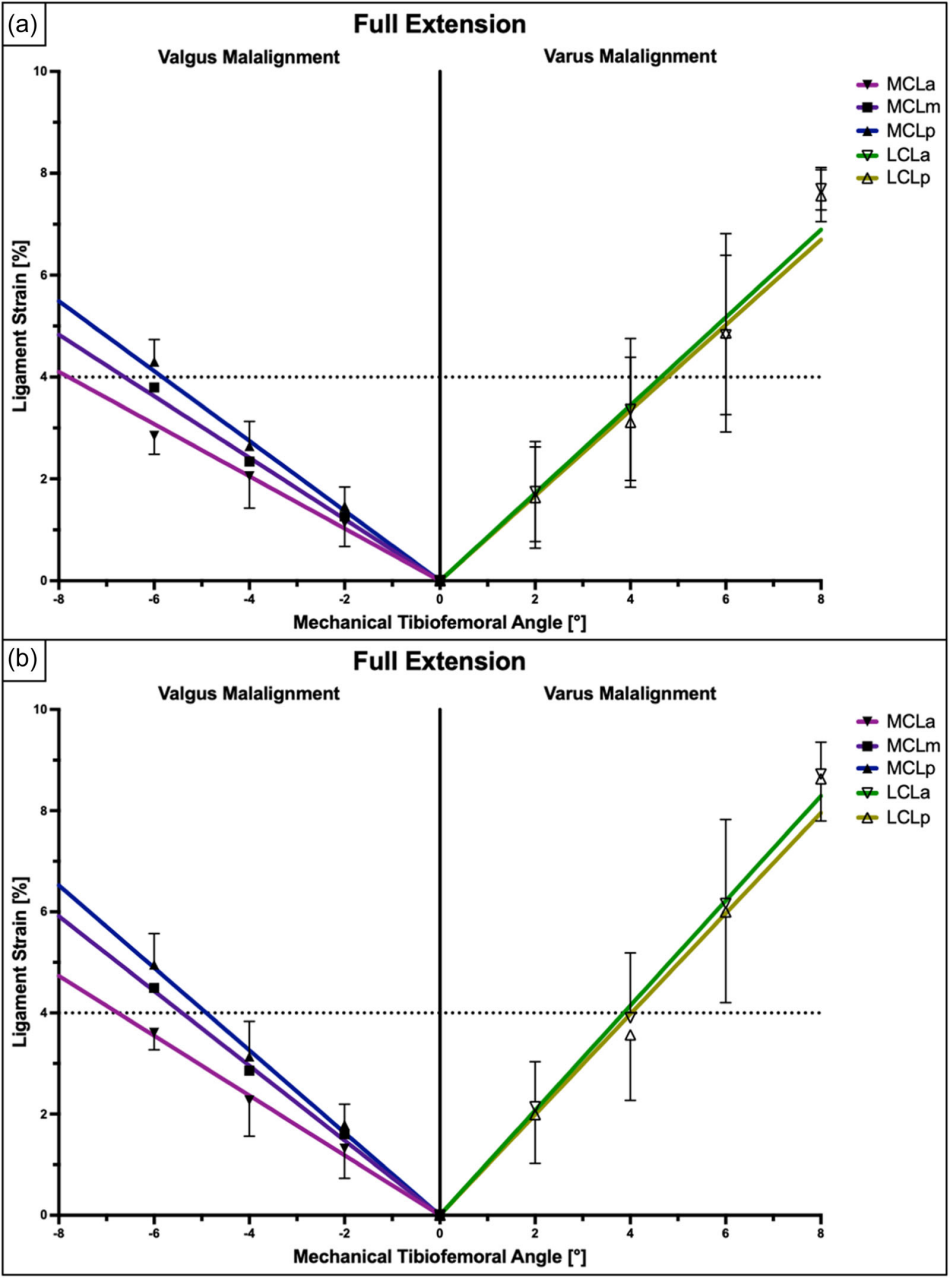
Ligament strain (in %) within different fibre regions of the superficial medial collateral ligament (sMCL) and lateral collateral ligament (LCL) at full knee extension relative to lower limb alignment presented as mean ± pooled standard deviations. Pearson's correlation analysis and linear regression showed a significant linear relationship between the mechanical tibiofemoral angle and collateral ligament strain under axial loads of 200 N (a) and 400 N (b). LCL, lateral collateral ligament; LCLa, anterior fibres of the LCL; LCLp, posterior fibres of the LCL; MCLa, anterior fibres of the sMCL; MCLm, middle fibres of the sMCL; MCLp, posterior fibres of the sMCNL.

**Figure 5:**
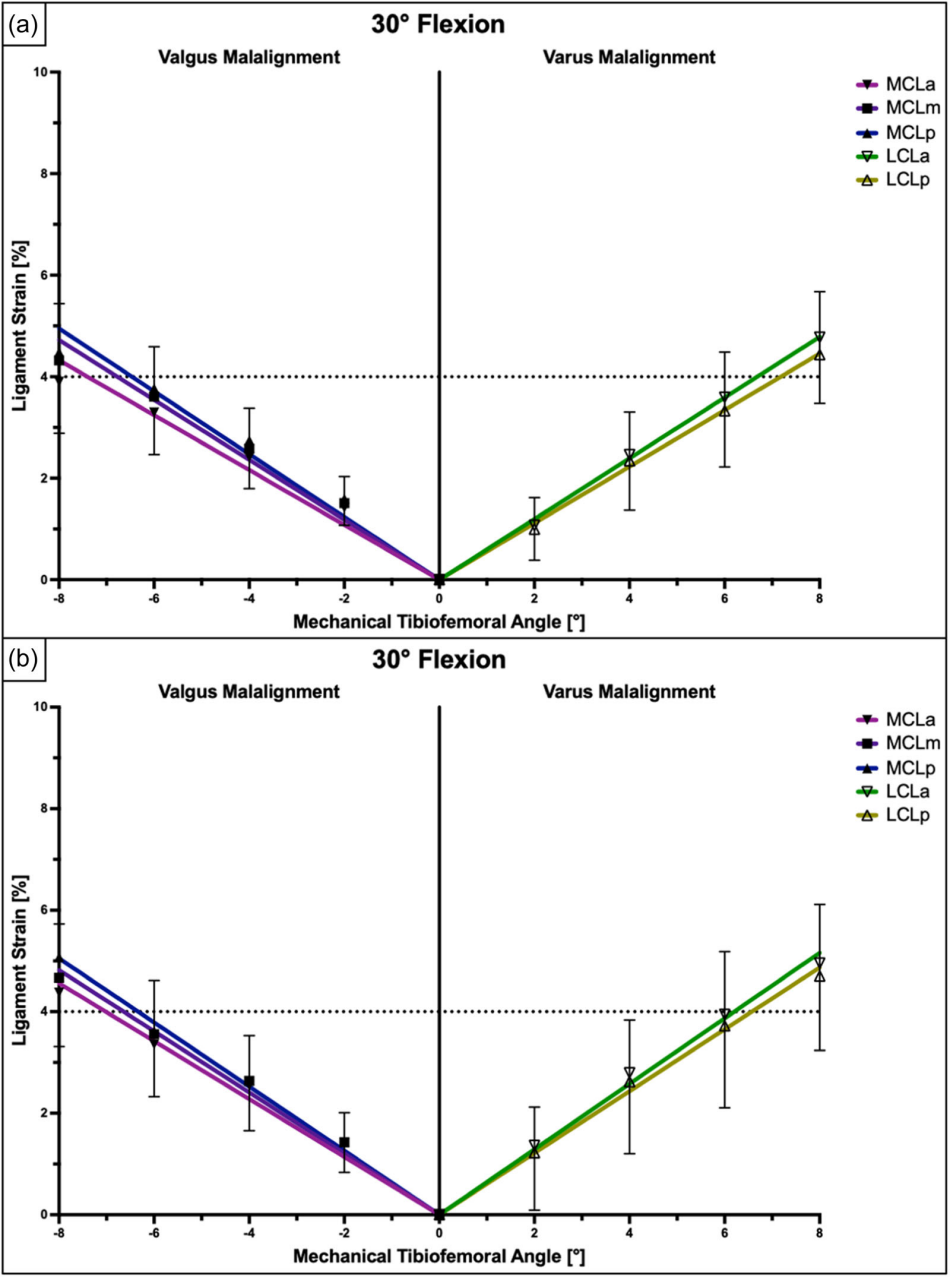
Ligament strain (in %) within different fibre regions of the superficial medial collateral ligament (sMCL) and lateral collateral ligament (LCL) at 30° of knee flexion relative to lower limb alignment presented as mean ± pooled standard deviations. Pearson's correlation analysis and linear regression showed a very strong and significant linear relationship between the mechanical tibiofemoral angle and collateral ligament strain under axial loads of 200 N (a) and 400 N (b). LCL, lateral collateral ligament; LCLa, anterior fibres of the LCL; LCLp, posterior fibres of the LCL; MCLa, anterior fibres of the sMCL; MCLm, middle fibres of the sMCL; MCLp, posterior fibres of the sMCL.

**Figure 6:**
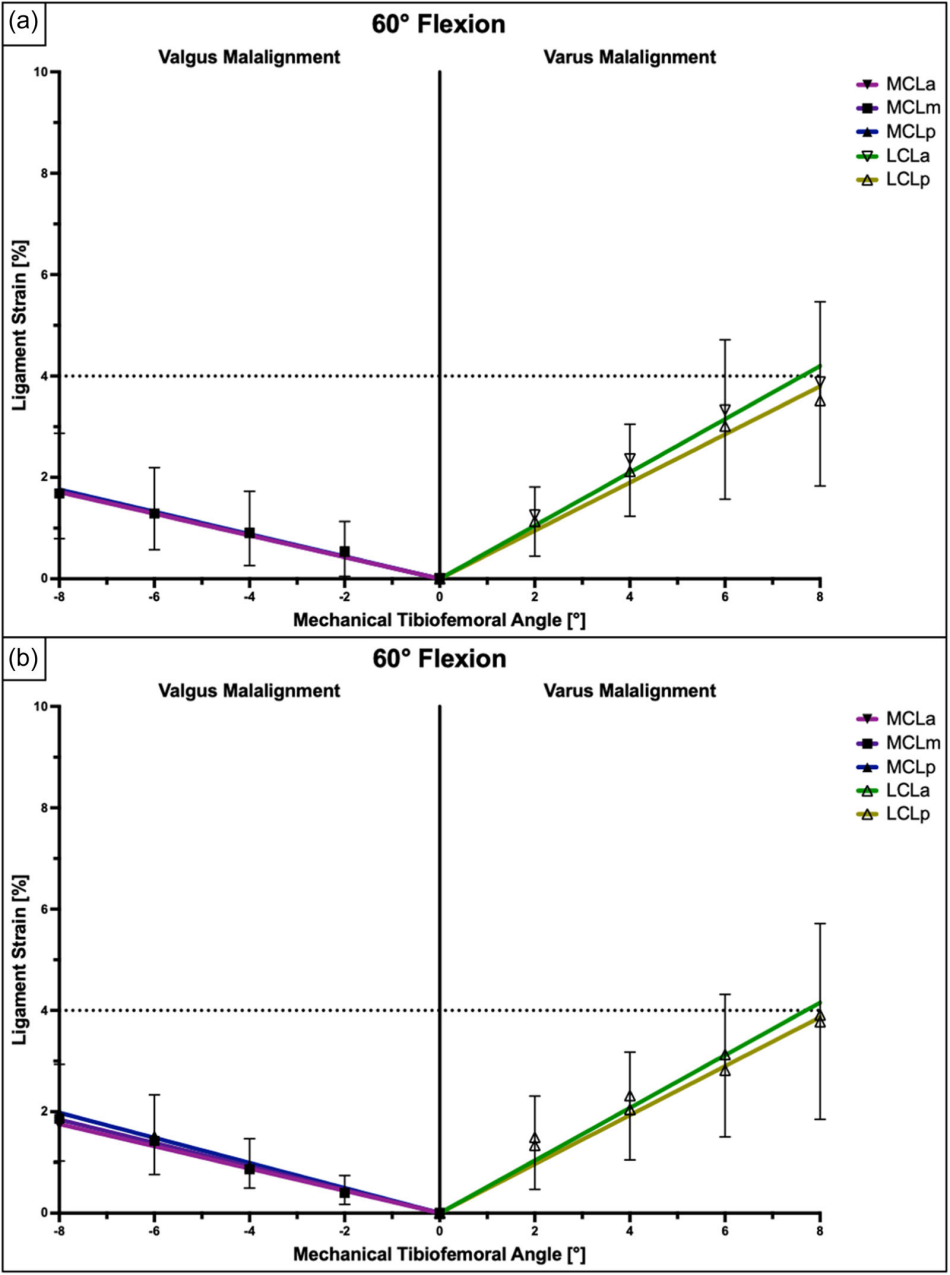
Ligament strain (in %) within different fibre regions of the superficial medial collateral ligament and lateral collateral ligament at 60° of knee flexion relative to lower limb alignment presented as mean ± pooled standard deviations. Pearson's correlation analysis and linear regression showed a very strong and significant linear relationship between the mechanical tibiofemoral angle and collateral ligament strain under axial loads of 200 N (a) and 400 N (b). LCL, lateral collateral ligament; LCLa, anterior fibres of the LCL; LCLp, posterior fibres of the LCL; MCLa, anterior fibres of the sMCL; MCLm, middle fibres of the sMCL; MCLp, posterior fibres of the sMCL.

The anterior and posterior LCL fibres showed the highest strain values at full extension, while the ligament strain gradually decreased with knee flexion (*p* < 0.001) (Table 1). Regardless of the amount of axial loading and knee flexion, ligament strain within the LCL showed a positive and linear relationship with the varus angle (Pearson's *r* > 0.98; *p* < 0.001). At full extension and 200 N of axial loading, the ligament strain within the anterior and posterior fibres of the LCL exceeded 4% at 4.6° and 4.8° of varus angulation, respectively. When 400 N of axial load was applied, 4% ligament strain was observed at 3.9° and 4.0° of varus angulation in the anterior and posterior fibres of the LCL, respectively (Figures 4, 5, 6 and Table 2).

**Table 1:** Ligament strain (in %) within the LCL depending on flexion angle and varus angulation presented as mean ± standard deviations.

Flexion angle (°)	mFTA (°)	Ligament strain at 200 N axial loading (%)		Ligament strain at 400 N axial loading (%)	
		LCLa	LCLp	LCLa	LCLp
0	0	0	0	0	0
	2	1.8 ± 1.0	1.7 ± 1.0	2.1 ± 0.9	2.0 ± 0.9
	4	3.4 ± 1.4	3.1 ± 1.3	3.6 ± 1.3	3.9 ± 1.3
	6	4.8 ± 1.6	4.9 ± 2.0	6.2 ± 1.7	6.0 ± 1.8
	8	7.7 ± 0.5	7.6 ± 0.5	8.7 ± 0.6	8.6 ± 0.8
30	0	0	0 0	0	0
	2	1.1 ± 0.7	1.0 ± 0.6	1.4 ± 1.3	1.2 ± 0.9
	4	2.5 ± 1.1	2.34 ± 1.0	2.8 ± 1.6	2.6 ± 1.2
	6	3.6 ± 1.4	3.3 ± 1.2	4.0 ± 1.8	3.7 ± 1.5
	8	4.8 ± 1.3	4.4 ± 1.2	5.0 ± 1.7	4.7 ± 1.4
60	0	0	0	0	0
	2	1.3 ± 0.8	1.1 ± 0.7	1.5 ± 1.0	1.3 ± 1.0
	4	2.4 ± 1.1	2.1 ± 0.9	2.3 ± 1.3	2.1 ± 1.1
	6	3.3 ± 1.8	3.0 ± 1.7	3. ± 1.63	2.8 ± 1.5
	8	3.9 ± 2.1	3.5 ± 1.5	3.9 ± 1.7	3.8 ± 1.9

Abbreviations: LCL, lateral collateral ligament; LCLa, anterior fibres of the LCL; LCLp, posterior fibres of the LCL.

**Table 2:** Mechanical tibiofemoral angle (in °) at 4% ligament strain.

Axial loading	Flexion angle (°)	mFTA (°) at 4% ligament strain				
		MCLa	MCLm	MCLp	LCLa	LCLp
200 N	0	−7.8	−6.6	−5.8	4.6	4.8
	30	−7.4	−6.8	−6.5	6.7	7.2
	60	−18.7	−18.6	−18.1	7.6	8.3
400 N	0	−6.8	−5.4	−4.9	3.2	4.0
	30	−7.0	−6.6	−6.5	6.2	6.6
	60	−18.2	−17.3	−16.1	7.6	8.1

*Note*: Strain levels associated with microdamages to the LCL and sMCL were observed at 4° varus (positive values) and 5° valgus (negative values) angulation, respectively.

Abbreviations: LCL, lateral collateral ligament; LCLa, anterior fibres of the LCL; LCLp, posterior fibres of the LCL; MCLa, anterior fibres of the sMCL; MCLm, middle fibres of the sMCL; MCLp, posterior fibres of the sMCL; mTFA, mechanical tibiofemoral angle; sMCL, superficial medial collateral ligament.

### Effect of valgus malalignment

The ligament strain within the sMCL showed a direct and linear association with the valgus angle (Pearson's *r* > 0.97; *p* < 0.001) (Figures 4, 5, 6). Dynamic valgus caused the highest strain values at full knee extension, while the ligament strain gradually decreased with knee flexion (*p* < 0.001) (Table 3). At full extension and 200 N of axial loading, the ligament strain within the anterior, middle and posterior fibres of the sMCL exceeded 4% at 7.8°, 6.6° and 5.8° of valgus angulation, respectively. When 400 N of axial load was applied, 4% ligament strain was measured at 6.8°, 5.4° and 4.9° of valgus angulation in the anterior, middle and posterior parts of the sMCL, respectively (Figures 4, 5, 6 and Table 2).

**Table 3:** Ligament strain (in %) within the sMCL depending on flexion angle and varus angulation presented as mean ± standard deviations.

Flexion angle (°)	mFTA (°)	Ligament strain at 200 N axial loading (%)			Ligament strain at 400 N axial loading (%)		
		MCLa	MCLm	MCLp	MCLa	MCLm	MCLp
0	0	0	0	0	0	0	0
	−2	1.1 ± 0.5	1.3 ± 0.5	1.5 ± 0.4	1.3 ± 0.6	1.6 ± 0.5	1.8 ± 0.4
	−4	2.1 ± 0.6	2.3 ± 0.6	2.7 ± 0.5	2.3 ± 0.7	2.6 ± 0.7	3.1 ± 0.7
	−6	2.9 ± 0.4	3.8 ± 0.8	4.3 ± 0.4	3.6 ± 0.3	4.5 ± 0.7	5.0 ± 0.6
	−8	NA	NA	NA	NA	NA	NA
30	0	0	0	0	0	0	0
	−2	1.4 ± 0.3	1.5 ± 0.4	1.6 ± 0.4	1.4 ± 0.6	1.4 ± 0.6	1.4 ± 0.6
	−4	2.4 ± 0.6	2.6 ± 0.6	2.8 ± 0.7	2.6 ± 0.9	2.6 ± 0.9	2.7 ± 0.9
	−6	3.3 ± 0.8	3.6 ± 0.7	3.8 ± 0.8	3.4 ± 1.1	3.6 ± 1.0	3.6 ± 1.0
	−8	3.9 ± 1.0	4.3 ± 0.9	4.5 ± 1.0	4.4 ± 1.1	4.7 ± 0.9	5.1 ± 0.7
60	0	0	0	0	0	0	0
	−2	0.5 ± 0.5	0.5 ± 0.5	0.6 ± 0.6	0.4 ± 0.2	0.4 ± 0.2	0.4 ± 0.3
	−4	0.9 ± 0.7	0.9 ± 0.7	0.9 ± 0.8	0.9 ± 0.4	0.9 ± 0.4	1.0 ± 0.5
	−6	1.3 ± 0.7	1.3 ± 0.8	1.3 ± 0.9	1.4 ± 0.6	1.4 ± 0.7	1.5 ± 0.8
	−8	1.7 ± 0.9	1.7 ± 1.0	1.7 ± 1.2	1.7 ± 0.7	1.9 ± 0.9	2.0 ± 1.0

Abbreviations: MCLa, anterior fibres of the sMCL; MCLm, middle fibres of the sMCL; NA, not applicable; MCLp, posterior fibres of the sMCL; sMCL, superficial medial collateral ligament.

## DISCUSSION

The main finding of the present study was that the coronal alignment of the lower limb was linearly related to the strain within the knee collateral ligaments, which was especially observed at full extension and higher axial loads. The posterior fibres of the LCL were exposed to strain levels associated with potential microdamages to the ligaments of more than 4% at 3.9° varus angulation, while the sMCL showed corresponding strain values for its posterior fibres at 4.9° valgus alignment.

Although repair and reconstruction of lateral [12, 15, 27, 30] and medial collateral ligament complex [26, 31, 44, 47] injuries result in good patient‐reported outcomes and knee stability, failure rates have been high, with reported rates of persistent and recurrent instability ranging from 10%–37% [21, 30, 31, 32]. The reasons for these high failure rates may be due to untreated bony malalignment [46] as lateral [33, 38] or medial [28, 38] soft tissue reconstructions may be subjected to excessive tensile forces in these patients. This could result in a gradual failure over time with a resulting varus or valgus thrust [23, 28, 32, 34, 46]. In fact, Noyes et al. [32] postulated that 37% of their posterolateral corner reconstructions failed due an untreated varus malalignment of 4°–6°. In line with these results, recent expert consensus statements recommend to consider long limb radiographs in all cases of chronic instability to rule out any varus/valgus malalignment [6, 7, 14]. In case of a posterolateral corner laxity, 85% of the leading knee experts would perform a valgus producing osteotomy if a concomitant varus deformity is observed but without providing a specific cut‐off value when an osteotomy is actually indicated [7]. In 2021, a German consensus working group postulated that a valgus ≥5° should be corrected prior to, or simultaneously, with the reconstruction of the medial collateral ligament [14]. However, these recommendations lack scientific evidence because there are no clinical data or studies correlating ligament strain with lower alignment to define thresholds for combined realignment and reconstructive procedures.

In the present study, dynamic varus/valgus caused a linear strain increase within both the LCL (Pearson's *r* > 0.98; *p* < 0.001) and sMCL (Pearson's *r* > 0.97; *p* < 0.001). In fact, a varus of 4° and a valgus of 5° were associated with more than 4% of ligament strain and might therefore cause structural damages to the lateral and medial collateral ligaments, especially when reconstructing these with tendon grafts [42]. As in the present study collateral ligament strain increased with higher axial load, the effects of varus or valgus malalignment on the ligament strain may be even higher in vivo, considering that the knee is exposed to approximately three times the body weight during normal gait [1, 43].

Considering that strain levels of more than 4% might cause microdamages to ligaments and tendons [42], reducing the strain to lower levels might protect the graft and could therefore reduce the risk of failure. In a previous biomechanical study, a valgus producing high tibial osteotomy significantly decreased the varus and external rotatory laxity of posterolateral corner‐deficient knees under applied varus moments but without restoring the stability of the native knee [23]. Mehl et al. [28] demonstrated that dynamic valgus loading caused increased ACL forces, especially if the posteromedial corner was deficient. In these valgus‐aligned knees with medial collateral ligament deficiency, correction to a straight leg axis was more effective in decreasing the forces within the ACL than an isolated reconstruction of the posteromedial corner [28]. These findings suggest that realigning osteotomies might be a powerful tool to reduce the tensile forces acting on the lateral or medial side of the knee rather than a soft tissue ligament reconstruction. Consistent with these biomechanical data, Arthur et al. [2] have clinically demonstrated that a valgus producing high tibial osteotomy significantly improved the subjective knee function in patients with combined chronic posterolateral instability and varus malalignment. In fact, eight of 21 patients (38%) reported satisfaction with their results after the osteotomy and felt that a second‐stage ligament reconstruction might not be necessary. This relieving effect of a realigning osteotomy is further illustrated by a recent clinical study evaluating the in vivo elongation patterns of the collateral ligaments during different functional activities. Throughout complete cycles of level walking, downhill walking and stair descent, neither the middle fibres of the sMCL nor the LCL exhibited strain values of more than 2.8%, as the knees of the patients were neutrally aligned (mTFA <3°) [19]. Therefore, individualized treatment strategies with combined realignment and reconstructive procedures may be required to improve outcomes and reduce failures following knee reconstructive surgeries in cases of chronic instability accompanied by a coronary malalignment.

The present study had several limitations which should be considered before interpreting the results. First, the serial testing might have caused a progressive parallel aligning of the collagen fibres within the collateral ligaments and thus a loss of viscoelasticity in the cadaveric specimen. Furthermore, the present test setup only allowed the measurement of length changes relative to the length at full extension of the knee, whereas the absolute tensile strain could not be assessed. Therefore, a subtle overestimation or underestimation of the axis‐dependent strain values could have occurred [25]. Last, the applied axial loads did not exceed 400 N, which is much lower compared to the weight acting during a gait cycle [1, 43]. Therefore, the data of the present study might underestimate the strain in vivo so that the critical threshold value of coronal deformity leading to ultrastructural damages of the collateral ligaments or tendon autografts could be overestimated.

## CONCLUSION

The strain within the native LCL and sMCL was linearly related to coronal lower limb alignment. Strain levels associated with potential ultrastructural damages to the ligaments of more than 4% were observed at 4° of varus and about 5° of valgus malalignment, respectively.

When reconstructing the collateral ligaments, an additional realigning osteotomy should be considered in cases of chronic instability with a coronal malalignment exceeding 4°–5° to protect the graft and potentially reduce failures.

## AUTHOR CONTRIBUTIONS

**Christian Peez**: Conception and design; testing and data acquisition; statistical analysis; writing. **Luise M. Hägerich**: Testing and data acquisition; writing. **Felix Ruhl**: Testing and data acquisition; internal review. **Matthias Klimek**: Conception and design; testing and data acquisition. **Thorben Briese:** Internal review. **Johannes Glasbrenner**: Internal review. **Adrian Deichsel**: Internal review. **Michael Raschke**: Internal review. **Christoph Kittl:** Writing; internal review. **Elmar Herbst**: Conception and design; testing and data acquisition; statistical analysis; writing.

## CONFLICT OF INTEREST STATEMENT

Elmar Herbst is deputy editor‐in‐chief for the Knee Surgery, Sports Traumatology and Arthroscopy (KSSTA). The remaining authors declare no conflict of interest.

## ETHICS STATEMENT

The specimens were dissected and biomechanically tested under the necessary permission from the Institutional Ethics Committee of the University of Muenster (File number 2020‐181‐f‐S).

## Data Availability

Data are available from the corresponding author upon reasonable request.
